# Manipulation of the diet–microbiota–brain axis in Alzheimer’s disease

**DOI:** 10.3389/fnins.2022.1042865

**Published:** 2022-11-04

**Authors:** Daniel Lee, Virginia M-Y. Lee, Seong Kwon Hur

**Affiliations:** ^1^Middleton High School, Middleton, WI, United States; ^2^Department of Pathology and Laboratory Medicine, Perelman School of Medicine, University of Pennsylvania, Philadelphia, PA, United States; ^3^Center for Neurodegenerative Disease Research, Perelman School of Medicine at the University of Pennsylvania, Philadelphia, PA, United States; ^4^Department of Neuroscience, Genentech, Inc., South San Francisco, CA, United States

**Keywords:** Alzheimer’s disease, tau, inflammation, gut–brain axis, probiotic, Mediterranean diet, Western diet

## Abstract

Several studies investigating the pathogenesis of Alzheimer’s disease have identified various interdependent constituents contributing to the exacerbation of the disease, including Aβ plaque formation, tau protein hyperphosphorylation, neurofibrillary tangle accumulation, glial inflammation, and the eventual loss of proper neural plasticity. Recently, using various models and human patients, another key factor has been established as an influential determinant in brain homeostasis: the gut–brain axis. The implications of a rapidly aging population and the absence of a definitive cure for Alzheimer’s disease have prompted a search for non-pharmaceutical tools, of which gut-modulatory therapies targeting the gut–brain axis have shown promise. Yet multiple recent studies examining changes in human gut flora in response to various probiotics and environmental factors are limited and difficult to generalize; whether the state of the gut microbiota in Alzheimer’s disease is a cause of the disease, a result of the disease, or both through numerous feedback loops in the gut–brain axis, remains unclear. However, preliminary findings of longitudinal studies conducted over the past decades have highlighted dietary interventions, especially Mediterranean diets, as preventative measures for Alzheimer’s disease by reversing neuroinflammation, modifying the intestinal and blood–brain barrier (BBB), and addressing gut dysbiosis. Conversely, the consumption of Western diets intensifies the progression of Alzheimer’s disease through genetic alterations, impaired barrier function, and chronic inflammation. This review aims to support the growing body of experimental and clinical data highlighting specific probiotic strains and particular dietary components in preventing Alzheimer’s disease *via* the gut–brain axis.

## Introduction

Lifestyle changes, particularly one’s diet, have emerged as a topic of significant interest in epidemiological research due to the capacity to modulate molecular mechanisms in the gastrointestinal tract, which has been associated with gastrointestinal and extra-gastrointestinal diseases (Vijay and Valdes, 2022). Recently, numerous studies have established the bidirectional communication between the gut microbiota and the central nervous system (CNS), described as the microbiota–gut–brain axis (GBA), and its potential effects in the context of neurological diseases, a reasonable connection to be made considering probiotics’ ability to restore gut homeostasis and serve as a host ameliorative factor (Yan and Polk, 2020; Socała et al., 2021). The rising interest in potential mediators between the gut microbiota and the pathogenesis of chronic neurodegenerative diseases, especially Alzheimer’s disease (AD), is associated with the public health and socioeconomic problem represented by neurodegenerative diseases, a concern exacerbated by a lack of concrete knowledge on the subject (Bredesen, 2016; Amato et al., 2019).

Dementia has been broadly defined as a loss of cognitive and behavioral functioning combined with an acquired memory impairment to an extent where a person’s ability to address daily living activities is diminished (Eschweiler et al., 2010; Zmily and Abu-Saymeh, 2013; Jithesh et al., 2020). The rapidly aging population and the consequent increase in the prevalence of dementia cases (Dua et al., 2017) are expected to present both immediate and long-term socioeconomic implications in the next few decades if no treatments are developed within that timeframe (Alzheimer’s Dementia, 2020).

At the center of focus is AD, a progressive disease beginning with mild memory loss to severe cognitive decline, accounting for 60–80% of all dementia cases (Garre-Olmo, 2018). AD is characterized by a complex neuropsychological profile consisting of interactions between different conditions (Ngandu et al., 2015), including age and the impairment of physiological barriers (Hebert et al., 2013), the inheritance of the APOE-4 gene (Seshadri et al., 1995; Farrer et al., 1997; Green et al., 2002), amyloid β (Aβ) peptide deposition (Fagan et al., 2006; Tapiola et al., 2009; Lee et al., 2019), intracellular neurofibrillary tangles (NFTs) (Franzmeier et al., 2020; Mattsson-Carlgren et al., 2020; Johansson et al., 2021), and external risk factors or conditions (Reitz et al., 2011; Beydoun et al., 2014). The public costs and consequences of the Alzheimer’s epidemic have been underestimated (Eschweiler et al., 2010), and major social trends will have direct and adverse effects on our ability to deal with this inevitable crisis in the years ahead.

Despite advances in our understanding of the pathological factors of AD at the cellular level, there have been no novel disease-modifying clinical therapies that have successfully addressed these identified mechanisms or that have effectively reversed the progression of AD. This includes Biogen’s anti-Aβ monoclonal antibody aducanumab, which was recently approved by the US Food and Drug Administration, aiming to remove amyloid protein depositions from the brains of patients at the expense of potential adverse effects (Ferrero et al., 2016; Yiannopoulou and Papageorgiou, 2020; Crosson et al., 2021; Hershey and Tarawneh, 2021). The evidence for any clinical benefit from aducanumab and previous drugs directed at amyloid deposits has been highly speculative based on both analyses of conflicting clinical trial data (Nicoll et al., 2019; Crosson et al., 2021; Knopman et al., 2021; McCleery and Quinn, 2021; Richard et al., 2021) and the compromised validity of the evidence presented due to the decision to approve the drug based on a surrogate endpoint, specifically Aβ reductions, whose status as a likely predictor of clinical benefit is also highly tentative (McCleery and Quinn, 2021). As most AD cases are late-onset and appear to have relatively long asymptomatic prodromal phases, novel therapies targeting the predicted inducers of AD may have a limited effect, especially when treatment is initiated in a patient who is already exhibiting symptoms (Golde, 2009). Thus, due to aducanumab’s inability to establish efficacy (Howard and Liu, 2020) and the persisting linear, amyloid-centric approach to AD research (Mullane and Williams, 2013), we must focus on new approaches that effectively target the disease trigger.

It is crucial to concentrate research efforts on identifying the precise stages of the complex pathological cascade that result in neuronal death in AD and, ideally, determining the relative sequence and timing of those events within that cascade (Golde, 2009). Subsequently, we will need to determine which of these steps are potentially targetable, develop novel therapies that interfere with these steps while considering the extent of disease progression (Lee et al., 2019), and continuously demonstrate that these therapies are effective in slowing or reversing AD (Golde, 2009; Sahoo et al., 2018; Salloway et al., 2020).

Despite the extensive research dedicated to deciphering AD pathogenesis and discovering novel drug treatments (Lee et al., 2019), the physiological complications established by a dynamic, selective barrier known as the blood–brain barrier (BBB) make it difficult to assess the therapeutic effectiveness of these methods due to restrictions on drug transportation (Fong, 2015; Pardridge, 2020), inadequate AD drug delivery models (Wan et al., 2009; de Lange and Hammarlund-Udenaes, 2015), and uncertainty of intracellular entry levels of the agent when measured through cerebrospinal fluid (CSF) (de Lange and Hammarlund-Udenaes, 2015). Furthermore, it has become clear that the involvement of pathological proteins in AD is incredibly intricate (Spires-Jones et al., 2017), and the complexity of clinical trial design has further complicated the consideration of combination therapies (Salloway et al., 2020). As a result, the gut microbiome has attracted attention as an alternative targetable area due to its ability to be manipulated through various nutritional therapies and indirectly interact with the BBB (Suganya and Koo, 2020). Several microbial-derived neurochemicals involved in the gut–microbiota–brain crosstalk appear to be implicated in the basis of neurodegeneration (Parker et al., 2020). This review aims to assist in the growing recognition of pathways in the GBA and their potential usefulness in addressing AD among medical communities. The exploration of this bidirectional communication, as well as the overall modulation process of one’s diet, will reveal new scenarios in chronic neurodegeneration research.

## Alzheimer’s disease neuroimmunology

AD is a neurodegenerative brain disorder characterized by a progressive decline in cognitive functions and reduced autonomy through the deterioration of neuronal cells and their networks (Kodis et al., 2018; Theofilas et al., 2018; Angelucci et al., 2019). While the pathophysiological conditions of AD are still relatively unclear, the disease has been attributed to extracellular accumulations of abnormally folded amyloid β (Aβ) proteins that polymerize and aggregate into plaques (Koffie et al., 2009; Wei et al., 2010; Hong et al., 2016; Jack et al., 2018; Jutten et al., 2019; Tiwari et al., 2019) through the alteration of APP (Herms et al., 2004; Ashley et al., 2005), as well as the hyperphosphorylation of the microtubule-associated protein tau into paired helical filaments and neuropil threads (Weingarten et al., 1975; Ballatore et al., 2007; Alavi Naini and Soussi-Yanicostas, 2015; Murray et al., 2015). This polymerization of proteins results in cytoskeleton disorganization in the axonal process (Himmelstein et al., 2012; Kolarova et al., 2012) and is thought to activate microglial cells, which further contributes to neurotoxicity (Rajmohan and Reddy, 2017; Ismail et al., 2020). In addition to genetic predispositions (Chakrabarti et al., 2015; van der Lee et al., 2018; Lee et al., 2020), environmental factors likely help trigger the onset of AD cases (Killin et al., 2016; Armstrong, 2019).

### The two-peak hypothesis

Despite the wide variation in clinicopathologic study designs and disagreement regarding amyloid pathology metrics, several ubiquitous findings identify a rather weak correlation between the density of amyloid plaques and the severity of cognitive decline compared to NFTs, especially in late prodromal phases (Markesbery et al., 2006; Jack et al., 2009; Nelson et al., 2009; Ismail et al., 2020). These observations indicate that simply suppressing Aβ peptide aggregation does not serve as a protective measure against AD, and alternate physiological aberrations caused by crosstalk between external systems (namely the GBA) may play an influential role.

### Cell-to-cell transmission of tau

Understanding tau dysfunctions and aggregation mechanisms are critical for developing new therapeutic strategies, especially considering the spread of tau toxicity through cell–to–cell transmission properties reported between anatomically connected brain regions (Uemura et al., 2020). To date, the exact mechanisms involved in the transmission of tau remain obscure, but it appears that misfolded forms of tau can serve as a template for the misfolding of normally structured tau (Jucker and Walker, 2013; Vijay and Morris, 2014) and spread trans-synaptically through afferent connections in correspondence with intrinsic connectivity (rather than proximity) (Ahmed et al., 2014), as well as in a manner reminiscent of prion spreading to induce the spread of filamentous tau (Hoenig et al., 2018; Schwarz et al., 2018). Macropinocytosis has been proposed as the most likely mechanism of tau uptake (Kerr and Teasdale, 2009; Holmes et al., 2013), delivering pathological tau in an exosome-dependent manner to unaffected cells and becoming internalized through the facilitation of microglia (Baker et al., 2016; Wang et al., 2017a), astrocytes, and oligodendrocytes (Martini-Stoica et al., 2018; Perea et al., 2019).

Similar to tau transmission throughout the CNS, tau could be delivered between different organs through neuronal connections (Zaccai et al., 2008). Considering the similar putative mechanisms of tau and α-synuclein (αSyn) transmission (Xu et al., 2014), whose pathology may be triggered by inflammatory agents in the enteric nervous system and spreads to the brain *via* the vagus nerve (VN) (Holmqvist et al., 2014; Sampson et al., 2016), tau pathology may be induced outside of the CNS. Notably, several tau isoforms were found to readily and bidirectionally cross the BBB in mice (Hidalgo et al., 2018), and tau was found in exosomes isolated from CSF (Saman et al., 2012) and the blood of patients with AD (Fiandaca et al., 2015). Thus, the cell–to–cell transmission of abnormal proteins that occur within the GBA deserves special attention in order to identify new targetable areas for AD therapies.

### Neuroinflammation

Research indicates that chronic inflammation through the build-up of ineffective glial cells and mediators from potentially long-distance paths of transmission (Jiang and Bhaskar, 2020) are implicated in AD pathogenesis. There are four types of glial cells in the CNS, two of which are focused on in this review as neuroinflammatory mediators between the gut and the brain: (1) microglia, which are phagocytic scavenger cells similar to macrophages in the nervous system (Keren-Shaul et al., 2017), and (2) astrocytes, which are starlike cells that comprise the most abundant proportion of glial cells in the brain (Jo et al., 2014).

#### The role of microglia in Alzheimer’s disease

Microglia, the first line of cellular defense, serve to dynamically survey the environment, sculpt neuronal circuits, and coordinate the communication between the innate and adaptive immune systems (Keren-Shaul et al., 2017). However, microglia-mediated responses are commonly described as “double-edged” because of their harmful side effects (Trotta et al., 2018), including the increase in localized cytokine concentrations (Keren-Shaul et al., 2017; Kwon and Koh, 2020), downregulation of Aβ and APP phagocytosis receptors (Ghosh et al., 2013; Brosseron et al., 2014; Kwon and Koh, 2020), and the tendency to sustain toxic recruitment around plaques. Furthermore, in conditions of gut dysbiosis, pathogenic compounds activate soluble myeloid cells 2 (TREM2), which are highly expressed in microglia (Lill et al., 2015; Reigstad et al., 2015; Piccio et al., 2016; Kwon and Koh, 2020), to impair bidirectional communication between the brain and the blood, the VN, or the glymphatic system (Natale et al., 2019). Thus, further studies are warranted to investigate the clinical benefits of targeting the microglial state at specific periods along the inflammatory cascade between the gut and brain.

#### The role of astrocytes in Alzheimer’s disease

In a healthy CNS, astrocytes are fundamental in maintaining homeostasis by clearing debris, protecting against oxidative stress, and presenting antigens for T cells (Constantinescu et al., 2005; Perea et al., 2009; González-Reyes et al., 2017). Astrocytes also play a key function in preserving the integrity of the BBB, a highly selective semipermeable border that regulates the movement of molecules, ions, and cells between the blood and the CNS (Daneman and Prat, 2015). Astrogliosis (Zamanian et al., 2012; Batarseh et al., 2016; González-Reyes et al., 2017; Liddelow et al., 2017; Fang et al., 2018; Guttenplan et al., 2020) stimulates the release of vasoactive endothelial growth factors, which increases BBB permeability and encourages the extravasation of leukocytes (Bush et al., 1999; Forman et al., 2004; Haseloff et al., 2005; Argaw et al., 2012; Cabezas et al., 2014; Sofroniew, 2015). NF-κB and complement signaling are also activated (Liao et al., 2004; Kawahara et al., 2009; Lian et al., 2015; Turillazzi et al., 2016; Jones and Kounatidis, 2017), impeding communication between neurons and glial cells through calcium and glutamate dysregulation (Rothstein et al., 1996; Berliocchi et al., 2005; Dabir et al., 2006; Friedman, 2006; Shigetomi et al., 2008; Ndountse and Chan, 2009; Sompol et al., 2017; Mahmoud et al., 2019), and producing inflammatory mediators in response to scavenger receptors ligands (Murgas et al., 2012) and LPS (Forloni et al., 1997), highlighting the gut microbiota’s influence on the immune system.

#### Perspectives on glial cell recruitment

**FIGURE 1:**
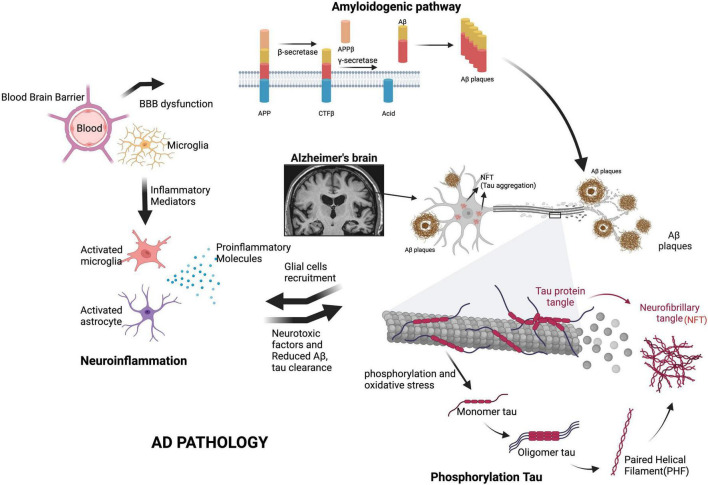
Key factors in AD pathogenesis. The formation of amyloid plaques through the overexpression of APP, tau hyperphosphorylation and NFT pathology, neuroinflammation, mitochondrial dysfunction and oxidative stress, translocation of harmful gut substances through a permeable BBB, and the eventual loss of synaptic function are the main causes of AD. Additional glial cell recruitment by proinflammatory mediators dysregulates Aβ and tau clearance, further contributing to neurodegeneration. Aβ, amyloid beta; APP, amyloid precursor protein; CTFβ, neurotoxic C-terminal fragment; BBB, blood-brain barrier. We have obtained the appropriate licenses from BioRender for this figure.

Extensive review has established the positive feedback loop between the functional changes in the Aβ-clearing mechanisms of both microglia and astrocytes as well as the promotion of neuroinflammatory mediators (Meda et al., 1995; Flint et al., 2008; Oksanen et al., 2017). The actions of several interwoven factors in AD pathophysiology are outlined in Figure 1. However, further studies are necessary to investigate why Aβ keeps accumulating despite the initial recruitment of supposedly protective cells in the context of external stresses on the GBA and to better understand the dynamic relationships between microglia, astrocytes, and the BBB.

## Mediators between gut dysbiosis and the brain in Alzheimer’s disease

### The gut microbiota

The human gastrointestinal (GI) tract accommodates a complex and dynamic community of microorganisms that regulate several aspects of host physiology and interactions with the environment (Thursby and Juge, 2017). The vast estimate of bacterial cells in the GI tract replaces various functions of the host, including the reinforcement of gut integrity, immunomodulation, vitamin synthesis, fermentation of food components into absorbable metabolites, and protection against pathogens (Heintz-Buschart and Wilmes, 2018). Thus, a shift in the bacterial composition produces adverse yet foreseeable results, including the pathogenesis of several notable inflammatories and neuropsychiatric disorders (Kho and Lal, 2018). Furthermore, dysbiosis of the gut microbiota is associated not only with intestinal disorders but also with numerous extraintestinal diseases, such as neurological disorders (Brandscheid et al., 2017), including AD, which is the focus of this review.

The composition of the microbiota is constantly being modified by the host and environmental factors, therefore, a method for regulating exposure to the host immune system is required. To fulfill this condition, the GI tract has developed a dynamic intestinal barrier consisting of several factors that rely on the gut microbiota, including physical (the epithelial and mucus layers), biochemical (antimicrobial enzymes), and immunological (Immunoglobulin A) components (Hooper and Macpherson, 2010; Thursby and Juge, 2017; Ghosh et al., 2021). Of the several bacterial phyla present in the gut microbiota, Firmicutes and Bacteroidetes account for 90%, with the rest composed of the subdominant phyla Proteobacteria, Actinobacteria, Fusobacteria, and Verrucomicrobia (Hill J.M. et al., 2014; Rinninella et al., 2019).

The Firmicutes phylum, which consists of mostly Gram-positive bacteria and makes up the largest portion of bacteria in the gut, is composed of over 200 different genera, such as *Lactobacillus, Eubacterium, Enterococcus, Ruminococcus*, and most notably *Clostridium* (Rinninella et al., 2019). Bacteroidetes are the second most abundant phyla of Gram-negative bacteria and consist of predominant genera, including *Bacteroides, Prevotella*, and *Xylanibacter* (Sánchez-Tapia et al., 2019). Other relevant genera present in the gut microbiota include *Escherichia* and *Desulfovibrio* from the Proteobacteria phylum and Actinobacteria (Pushpanathan et al., 2019). The unique gut microbiota profile of enterotypes (Rinninella et al., 2019) will remain relatively stable after reaching a homeostatic climax composition of appropriate diversity, but is heavily affected by age, in which the microbiota composition becomes less diverse due to the characterization of higher Firmicutes: Bacteroidetes ratio, an increase in Proteobacteria and a decrease in *Bifidobacterium* (Ottman et al., 2012). Similar to age, prominent metabolic diseases establish a similar association between reduced microbial diversity and poor health (Valdes et al., 2018). Thus, diversity seems to be a good indicator of a healthy gut, accentuating the role of dietary patterns in cognitive health.

#### Gram-positive bacteria

Studies on the metagenomic sequencing of the human distal gut microbiome have revealed that a healthy gut microbiome is predominantly composed of *Clostridium* and *Lactobacillus* species with antimicrobial characteristics (Sweeney and Morton, 2013). Bacteria with a Gram-positive cell wall structure, such as *Lactobacillus Rhamnosus* and *Bifidobacterium*, can produce a favorable assortment of short-chain fatty acids (SCFA), most notably butyrate, by fermenting nondigestible carbohydrates and dietary fibers (Huang et al., 2018; Dalile et al., 2019).

##### Short-chain fatty acids

SCFAs are involved in plant-based food consumption and are associated with mucus production, the promotion of intestinal barrier integrity, and limits the translocation of bacterial proinflammatory molecules (Fukuda et al., 2011; Chambers et al., 2018; Silva et al., 2020). SCFAs are thought to play a critical role in the gut–brain crosstalk through brain uptake and modulation (Duscha et al., 2020), the inducement of morphological changes in glial cells, providing energy for colonocytes, and supporting the integrity of the BBB (den Besten et al., 2013; Silva et al., 2020). SCFA absorption by colonic epithelial cells also modifies the luminal pH levels in the colon, preventing pH-sensitive pathogenic bacteria associated with AD (Yatsunenko et al., 2012; Hill J.M. et al., 2014; Chakraborti, 2015). Intelligent pharmacological approaches consisting of anti-inflammatory drugs (Hill J.M. et al., 2014) in combination with dietary strategies that encourage the adequate production of SCFAs could be useful in the clinical management of AD.

#### Gram-negative bacteria

Bacteroidetes is a phylum of Gram-negative rod-shaped bacteria that account for about 25% of the anaerobes residing in the human colon (Wexler, 2007; Johnson et al., 2017). Sequence-based studies of gut bacterial diversity have revealed that *Prevotella* is more common in a plant-rich diet and has been associated with vegetarianism while the *Bacteroides* genus constitutes diets rich in animal protein and saturated fats (Tomova et al., 2019).

*Bacteroidales* and *Bacteroides* are by far the most abundant contributors to the biosynthesis of lipopolysaccharides (LPS), which are lipid-soluble outer-membrane complexes of Gram-negative bacteria (d’Hennezel et al., 2017; Tomova et al., 2019). Such endotoxins contribute to oxidative stress and the overproduction of inflammatory cytokines/chemokines through multiple structural components (Huszczynski et al., 2020) that initiate downstream inflammatory signaling, such as the NF-κB signaling pathway (Guo et al., 2013; Lin et al., 2020).

The translocation of endotoxins into the blood is accomplished by the breakdown of the intestinal epithelial barrier by pathogenic bacteria, specifically through interruptions in the paracellular, semipermeable barriers between individual epithelial cells composed of tight junction proteins (Ghosh et al., 2020; Hollander and Kaunitz, 2020). Increased paracellular transport of luminal bacteria and associated metabolites into the Lamina Propria and systemic circulation promotes proinflammatory interactions between bacterial products, epithelial cells, and immune cells activated through the TLR4/FAK/MyD88 signal transduction axis and auxiliary molecules associated with intracellular signaling (Guo et al., 2015; Lykhmus et al., 2016; Ghosh et al., 2020; Hollander and Kaunitz, 2020). Endotoxemia may be the basis for neurological complications in AD due to its role in gut dysbiosis and chronic inflammation.

#### The firmicutes: Bacteroidetes ratio

The relationship between Firmicutes and Bacteroidetes has become an auspicious target for the nutritional and therapeutic treatment of various pathological conditions affecting the GI tract, host energy metabolism, the immune system, and the CNS (Magne et al., 2020). Though the relative abundance of Firmicutes and Bacteroidetes varies between humans, abnormal alterations to the Firmicutes: Bacteroidetes ratio leads to gut dysbiosis and vice versa. Harach et al. (2017). have determined that there is a negative correlation between Firmicutes and Bacteroidetes: as one increases, the other decreases. Gut dysbiosis observed in this relationship specifically occurs either through the low-grade inflammation resulting from the release of lipid A (a component of LPS) by a surplus of Gram-negative bacteria or the increased efficiency of fermentation and metabolism of carbohydrates and lipids by an overabundance of Firmicutes bacteria (Bostanciklio, 2019; Stojanov et al., 2020).

##### Dysbiosis

Substantial studies have presented compelling evidence connecting impaired gut microbial diversity and abnormal Firmicutes: Bacteroidetes ratio to AD. In a study of 5XFAD mice with severe amyloid pathology (Oakley et al., 2006), it was noted that the abundance of Firmicutes greatly increased while the abundance of Bacteroidetes decreased, perhaps through the presence of metabolites belonging to other pathogenic bacteria (Brandscheid et al., 2017). Aβ and hyperphosphorylated tau levels in APPPS1 mice increased in correlation with age, thereby increasing neurodegeneration and reducing the abundance of Firmicutes, Verrucomicrobia, Proteobacteria, and Actinobacteria (Harach et al., 2017). Other studies that evaluated the diversity of Firmicutes and Bacteroidetes in patients with brain amyloidosis have determined reduced richness and diversity of bacterial taxa (Vogt et al., 2017) and a significant reduction in *Eubacterium rectale* and *Bacteroides fragilis* (Cattaneo et al., 2017), both of which have anti-inflammatory properties (Troy and Kasper, 2010; Islam et al., 2021). Furthermore, Zhuang et al. (2018) discovered a lower level of Bacteroidetes in patients with AD, while an increase in the *Ruminococcus* species, a Gram-positive bacterium with the potential to degrade mucus through the expression of intramolecular trans-sialidases (Mazloom et al., 2019).

Compared to cognitively normal controls, patients with AD had an altered gut microbiota composition due to differences in *Bacteroidales*, *Ruminococcaceae*, *Selenomonadales*, and *Lachnoclostridium* (Zhuang et al., 2018). Restoring and diversifying the *Firmicutes: Bacteroidetes* ratio with the proper probiotics and diet choices, especially through the introduction to a plant-based diet and probiotics, such as *Lactobacillus* (Mazloom et al., 2019), can help suppress inflammatory responses, enhance intestinal barrier function, and inhibit pathogenic bacterial adhesion, a strategy that has already shown promise in some patients (Tang et al., 2016; d’Hennezel et al., 2017). However, contradictory results concerning bacterial relationships remain (Chenoll et al., 2011), and further research is required to better understand these two dominant bacterial populations to choose the most appropriate probiotic strains or mixtures for optimal health outcomes.

#### Other prominent phlya

The population of Proteobacteria, a major phylum of Gram-negative bacteria, and Actinobacteria, a phylum of Gram-positive bacteria, typically constitute the remaining proportion of bacterial organisms in the gut microbiota (Khanna and Tosh, 2014). Actinobacteria, specifically *Bifidocterium*, are fundamental in the regulation of gut homeostasis and potentially improve memory function *via* hormonal signaling in the GBA (Mazloom et al., 2019). The effectiveness of such applications is possible due to the beneficial properties of *Bifidobacterium*, including the stimulation of dendritic cells to regulate intestinal immune homeostasis, the establishment of immune-protective measures while suppressing proinflammatory cytokines, and the overall improvement of GI barrier function (Mazloom et al., 2019).

Certain *Bifidobacteria*, in conjunction with *Lactobacilli*, have been observed to produce health-promoting conjugated linoleic acid and small antimicrobial peptides, which ward off pathogenic bacteria, such as *E. coli*, *Salmonella*, *H. pylori*, *Listeria monocytogenes*, and *Rotavirus* (Chenoll et al., 2011). In contrast, the increased abundance of Proteobacteria in the gut has been linked to gut dysbiosis and the pathogenesis of the disease (Rizzatti et al., 2017; Ruotolo et al., 2020). Although the connection between the prevalence of Proteobacteria in the gut during disease is not fully understood, it has been hypothesized that a combination of decreased oxygen levels by colonocytes in the lumen through beta-oxidation processes and the increased production of nitrate in the case of intestinal inflammation creates an anaerobic environment in which Proteobacteria can flourish (Rizzatti et al., 2017). Similar to *Bacteroidetes*, Proteobacteria also contain LPS in their outer membrane due to their Gram-negative staining, which induces low-grade inflammation and metabolic disorders (Azad et al., 2018).

#### Microbiome analysis

The microbial composition is thought to be influential in AD etiology and has attracted significant attention (He et al., 2020). Studies on the gut microbiota in patients with AD using fecal DNA samples revealed an overall decreased abundance of *Firmicutes* and *Actinobacteria* and an increase in Bacteroidetes and Proteobacteria (Rizzatti et al., 2017). Interestingly, the study identified potentially protective bacterial taxa in non-AD patients and observed a positive correlation between CSF inflammation biomarkers and an increased abundance of *Bacteroides* and *Clostridiaceae*, supporting a link between altered gut microbial composition and glial activation in AD (Rizzatti et al., 2017).

Similarly, a different study reported that a peripheral inflammatory state in AD patients with brain amyloidosis may be associated with an increase in proinflammatory *Escherichia/Shigella* and a decrease in anti-inflammatory *E. rectale* (Cattaneo et al., 2017). Although not as reliable, postmortem human studies do highlight the presence of various other pathogens, including *H. pylori*, *Chlamydophila pneumoniae*, and other spirochetes (Kowalski and Mulak, 2019). Additional studies on the microbiome of AP5P/PS1 transgenic mice revealed a general increase in *Helicobacteraceae*, *Desulfovibrionaceae*, *Rikenellaceae*, *Odoribacter*, and most notably *Proteobacteria* (typically from the *Betaproteobacteria* class) while showing a decrease in *Prevotella* (Biagi et al., 2010; Bäuerl et al., 2018). Mice, which are carriers of the APOE4 genotype, were associated with a loss of SCFAs due to a failure to replace decreased levels of butyrate-producing bacteria, such as *Prevotellaceae* and *Ruminococcaceae* (Tran et al., 2019).

### The role of the gut–brain axis in Alzheimer’s disease

It is crucial to identify the main mechanisms of communication between the brain and the gut to understand how modifications to the gut microbiota can affect the nervous system in the context of AD. The GBA describes a complex system of bidirectional communication between the cognitive centers of the brain, intestinal functions and permeability, immune activation, enteric reflex, and enteroendocrine signaling (Carabotti et al., 2015). The GBA provides various metabolic pathways by which each respective system can influence the other, through mechanisms, such as the immune system, microbial metabolites, tryptophan metabolism, the VN, and the enteric nervous system, as well as the hypothalamus–pituitary–adrenal (HPA) axis (Ait-Belgnaoui et al., 2012; Cryan et al., 2019). The regulation of not only these mechanisms but also the permeability of the BBB and the intestinal barrier ensure efficient communication between the gut microbiota and the brain, which is essential for protecting the CNS (Daneman and Prat, 2015).

The general disruption of any neural, hormonal, and immunological pathways that define the GBA will affect intestinal motility and secretion, resulting in visceral hypersensitivity and cellular alterations of the immune and enteroendocrine systems (Appleton, 2018). Functional GI disruptions have pathological consequences associated with gut-related metabolic disorders, stress-related psychiatric disorders, and brain-related disorders (Appleton, 2018). Considering the bidirectional influence of the GBA, as well as emerging evidence advocating the use of probiotics to alleviate conditions exacerbated by gut dysbiosis, alterations to the gut microbiota may improve AD conditions.

#### Regulators of the gut–brain crosstalk

For communication between the CNS and the gut microbiome to occur, the passage through the intestinal barrier and the BBB must be well-regulated (Zhu et al., 2020). A layer of epithelial cells and mucus protects the intestinal barrier, facilitating the translocation of microbes, bacterial products, and other metabolites into the systemic circulation and lymphatic tissue (Zheng et al., 2020). Prominent bacterial molecules, such as SCFAs and LPS are major influencers of this semipermeable barrier by either promoting or inhibiting the translocation of proinflammatory cytokines, affecting the body’s immune response (Jergens et al., 2021).

##### Blood-brain barrier

The BBB is a highly specialized barrier of endothelial cells and intercellular junctions that line cerebral microvessels, regulating the entry of plasma components, red blood cells, leukocytes, and potentially neurotoxic molecules from the plasma to CSF in the CNS (Fung et al., 2017). The breakdown of the BBB may occur due to aging, disease, infection, or genetic factors that may drive fibrin and CNS amyloid deposition through dysfunctional Aβ transport (i.e., decreased activity of Aβ clearance mechanisms while an increase in Aβ40 and RAGE activity) (Rothhammer et al., 2016), but the role of circulating immune system cells in AD and the mechanisms that regulate this breakdown of the BBB are still poorly understood (Zenaro et al., 2017). However, BBB dysfunction is a major cause of chronic neuroinflammation through reduced Aβ clearance and endothelial transport, impairment of pericyte functions, decreased integrity of tight junctions, accumulation of toxins in the CNS, and the recruitment of glial cells and leukocytes in the brain (Fung et al., 2017). Further insight into leukocyte-endothelial interactions in AD, adhesion molecules, and bacterial-linked low-grade inflammation in both the intestinal and the BBB may offer valuable biomarkers of AD.

##### The immune system

The immune system is one of the main pathways through which microbes of the gut microbiota can interact with the GBA and the CNS (Appleton, 2018). The immune system consists of a complex network of innate components that can adapt and respond to various microbial and environmental challenges, a system that has largely evolved to maintain the symbiotic relationship of the host with the highly diverse microbes of the gut (Zenaro et al., 2017). Most host-microbe interactions occur at the luminal-mucosal interface secreted by the single-cell layer of the gut, in which the exchange of molecules through the mucous layer and epithelium serves to facilitate communication between the gut and the immune system through the recognition of antigens (Profaci et al., 2020).

Along with the stimulation and activation of the innate and adaptive immune systems, emerging evidence indicates that several commensal bacteria metabolites, including neuromodulators, bacteriocins, bile acids, choline, and SCFAs, are immunomodulatory and produce neurotransmitters, neuropeptides, endocrine messengers, and microbial by-products that can enter the blood and lymphatic systems, influencing neural messages through afferent neurons (Profaci et al., 2020). Likewise, the increased expression of proinflammatory cytokines and circulating bacterial endotoxins (LPS) due to gut dysbiosis can modulate the GBA by increasing intestinal permeability and triggering chronic low-grade inflammation in extraintestinal areas of the body (Appleton, 2018). Based on this influence, the colonization of the gut microbiota with a specific balance of anti-inflammatory bacteria, such as *Bacteroides fragilis*, *Bifidobacterium*, and *Lactobacillus*, as well as pro-inflammatory bacteria, including, *E. coli*, *Enterobacteriaceae*, and other segmented filamentous bacteria may encourage varied immunological responses (Fung et al., 2017).

#### Microbial metabolites

##### Tryptophan

Microbial metabolites have demonstrated an active role in directly governing CNS physiology through neurotransmitters and can influence the function and maturation of microglial cells (Belkaid and Hand Timothy, 2014), as well as astrocytes, which are affected by tryptophan metabolism (Wang Y. et al., 2018). The gut microbiota can influence astrocytes by activating receptors through microbial metabolites, such as Type 1 interferon and indoxyl-3-sulfate, which display anti-inflammatory properties and improve symptoms in experimental autoimmune encephalomyelitis (Rothhammer et al., 2016; Fung et al., 2017). Gut microbes that metabolize the essential amino acid tryptophan may interfere with tryptophan metabolism, limiting tryptophan availability for the host, which affects serotonergic neurotransmission, the function of central and enteric nervous systems, and may result in impaired cognitive functions (Kaur et al., 2019). Additionally, tryptophan can be diverted away from serotonin production and into the kynurenine/quinolinate pathways (metabolites of tryptophan), which has been proposed to disturb neurological functions and cause depression-like symptoms due to the reduction of tryptophan concentrations in the blood, thus limiting the production of important neurotransmitters in the brain, such as serotonin (Kaur et al., 2019). The significance of tryptophan metabolism in the GBA and its corresponding bacterial pathways indicates the importance of neuroactive compounds in host-microbiota interaction.

##### Neuroactive compounds

The gut microbiota encourages the production, modulation, and release of essential neurotransmitters that influence immune cells and alter brain functioning. Various *Lactobacillus* (Strandwitz, 2018), *Bifidobacterium*, and *Escherichia* species can synthesize Gamma-aminobutyric acid (GABA), the major inhibitory neurotransmitter of the CNS that is distributed throughout the host (Cryan and Dinan, 2012). GABA secretion decreases intracellular pH *via* the glutamtic acid resistance system, and its production has been linked to insulin sensitivity and reduced sensitivity to visceral pain in rat models (Strandwitz, 2018). Additionally, GABA levels in the cortices and the hippocampal regions in the brain are thought to be involved in controlling cortical excitability and working memory performance, and the functional enhancement of GABA inhibitory interneurons may help establish a resistance to Aβ deposition (Mandal et al., 2017; Xu Y. et al., 2020b). Other examples of species that can produce neurotransmitters include *Candida*, *Streptococcus*, and *Escherichia* in the production of serotonin, *Saccharomyces* in the production of noradrenaline, *Lactobacillus* in the production of acetylcholine, and *Bacillus* in the production of dopamine (Halverson and Alagiakrishnan, 2020). Bacterial production of SCFAs, such as butyric acid, propionic acid, and acetic acid, also affects the release of mucosal serotonin by stimulating the sympathetic nervous system, demonstrating the broader influence microbial metabolites have on cognitive processes (Reigstad et al., 2015; Maltz et al., 2018).

##### Microbial amyloids

Microbial-generated amyloids may play a crucial role in neuroinflammation and the formation of plaques (Fulop et al., 2018). Fungal surface structures and amyloidogenic fungal proteins, *E. coli* and curli fibers, and various Gram-negative bacteria with their respective amyloid systems were discovered to facilitate surface adhesion and biofilm development (a matrix of extracellular polymeric amyloids and other lipoproteins), which placed a tremendous systemic amyloid burden on the host when the intestinal barrier and the BBB were restructured (Hill and Lukiw, 2015). Biofilm-associated amyloids and lipoproteins produced by Firmicutes, Bacteroidetes, and Proteobacteria are recognized by microglia, which subsequently induce the production of cytokines that drive NF-kB signaling, phagocytosis, and innate immune defense responses that are repeatedly recognized to exacerbate the inflammatory response through Aβ peptide production (Miller A. L. et al., 2021).

Other than contributions to the autoimmune process, it has been hypothesized that bacterial amyloids may indirectly induce the aggregation of human amyloidogenic proteins (Friedland, 2015; Chen et al., 2016; Miraglia and Colla, 2019). Bacterial amyloids, such as curli, share structural and physical properties with human pathogenic amyloids and activate the same toll-like receptors that recognize Aβ and αSyn, which are further propagated from the gut to the brain by bacterial amyloids in biofilm and may cause motor deficits in AD and PD (Kim et al., 2019). Notably, it has been argued that microbial products and signals from the gut, such as αSyn, reach the brain *via* the VN rather than the circulatory system and the BBB, as corroborated in some mice models (Challis et al., 2020). Regardless of how exactly bacterial amyloids affect the CNS in AD, probiotics and other gut-modulatory therapies have been proposed as precautionary measures to reduce the pathological amyloid content of both the gut and the brain and reverse amyloid-induced neuroinflammation (Naomi et al., 2021).

#### The vagus nerve

A key mechanism of communication in the GBA involves the VN, which is the longest cranial nerve in the body and the main component of the parasympathetic nervous system that sends bidirectional information through afferent and efferent fibers (Breit et al., 2018). Because of its role in interoceptive awareness, the VN can sense microbiota metabolites through its vagal afferents, which can detect chemicals absorbed across the epithelial layer or released by epithelial cells in response to luminal stimuli (Xu Y. et al., 2020a). In turn, this gut information is then transmitted to the CNS, where it is integrated into the central autonomic network and relayed in the brainstem, where gut vagal afferents mostly synapse onto neurons in the solitary tract (Bonaz et al., 2018; Suarez et al., 2018).

The stimulation of vagal afferent fibers in the gut influences monoaminergic brain systems and engages the hypothalamus and limbic system, designating the VN as an important coordinator between neural, behavioral, and endocrine responses as well as a key target for the treatment of psychiatric, neurological, and inflammatory diseases (Breit et al., 2018). Conversely, the CNS can influence the intestines *via* the VN and efferent fibers (Alfonsetti et al., 2022). A cholinergic anti-inflammatory pathway that can dampen peripheral inflammation and decrease intestinal permeability has been identified in the VN’s fibers, thus playing an indirect but significant role in the alteration of microbiota composition (Bonaz et al., 2018). Descending projections from brain systems that have been activated due to stress inhibit the VN, resulting in deleterious effects on the GI tract and the autonomic activities of the gut that are observed in gastrointestinal disorders (Bonaz et al., 2018). Considering the extent of the VN’s role in the modulation of inflammation, the regulation of nutrition, and overall microbiota–brain communication, it may be advantageous to monitor vagal tones through VN stimulation, nutritional approaches, and psychotropic drugs to address gut dysbiosis (Breit et al., 2018).

#### The hypothalamus–pituitary–adrenal axis

Afferent spinal and vagal sensory neurons in the VN carry signals from the intestinal end to the brain stem, which engages the hypothalamus system (Breit et al., 2018). The HPA axis is a major neuroendocrine system that responds to psychological and physical stressors by releasing corticotropin-releasing factor (CRF) and, subsequently, adrenocorticotropic hormone (ACTH), which elevates cortisol levels (Farzi et al., 2018).

The neuroendocrine processes of the HPA axis in the context of AD can result in the continued activation of the immune system and the HPA axis itself, which encourages further alterations to intestinal permeability and motility when combined with a microbiota-driven proinflammatory state and increased exposure to LPS (Farzi et al., 2018). The direct consequence of early dysregulation of the HPA axis in patients with AD resulted in glucocorticoid over-secretion, which was discovered to be highly toxic in limbic structures and increased AD biomarkers (Canet et al., 2019). The hyperactivity of the HPA axis, elevated serum cortisol levels, and the dysregulation of the negative corticosteroid feedback system can increase the vulnerability of cerebral neurons and disrupt critical neurotrophic functions (Carabotti et al., 2015), indicating that the cascade of glucocorticoids may be the major mechanism responsible for behavioral alterations during AD progression (Ahmad et al., 2019). In addition to hypercortisolemia, the dysfunction of the hypothalamic–pituitary–gonadal axis may affect hormones with hippocampal receptors, resulting in the dysregulation of neuronal development and function in AD (Ahmad et al., 2019).

The gut microbiota’s involvement in the HPA axis response to stress, and ultimately AD, is a prime target for therapeutic strategies aiming to reverse stress-related conditions of AD. Studies that observed exaggerated HPA axis responses to stress in GF mice and reduced responses in GF mice colonized with specific *Bifidobacterium* species suggest that exposure to continuous stress can alter the organism’s microbiota composition and that the introduction of specific microbial populations can influence an organism’s stress responsiveness (Foster et al., 2017). The role of a gut-influenced HPA axis in the reversal of AD remains to be elucidated, and more detailed studies are necessary to establish the relationship between the hypersecretion of adrenal cortisol and amyloid pathology (Ahmad et al., 2019).

### Perspectives on the gut–brain axis

**FIGURE 2:**
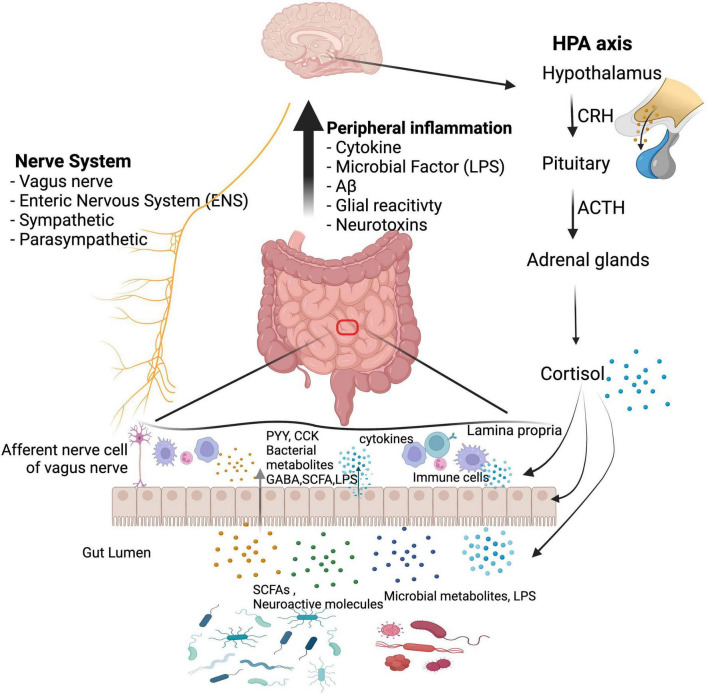
Communication networks between the gut microbiota and the brain. Communication pathways include immunological, neural, and hormonal pathways, which can function collectively: (1) MAMPs can activate both the peripheral and the central immune system; (2) microbial metabolites reach the brain to regulate neurological health; (3) a bidirectional influence is established between the CNS and the gut microbiota through the vagus nerve and the enteric nervous system; (4) interactions with the HPA axis regulate brain function and gut microbiota composition. Gut dysbiosis decreases the presence of beneficial substances (e.g., SCFAs) and increases toxic substances (e.g., amyloids and LPS), which cause the intestinal mucosal barrier and BBB to become more permeable, exacerbating inflammatory conditions. CRH, corticotropin-releasing hormone; ACTH, adrenocorticotropic hormone; PYY, peptide tyrosine tyrosine; CCK, cholecystokinin; GABA, gamma-aminobutyric acid; SCFA, short-chain fatty acid; LPS, lipopolysaccharide. We have obtained the appropriate licenses from BioRender for this figure.

The complexities of the GBA are not completely understood. However, changes in the gut–brain crosstalk that are induced by AD pathogenesis are generally associated with neurological complications and vice versa, as depicted in Figure 2. Considering that gut microbial disruptions induce alterations in the brain, it is within reason to expect a reversal of those modifications if the gut microbiota can be restored to its appropriate state (Boehme et al., 2020). Much of the observable influence of probiotics, fermented foods, and plant-based dietary patterns on cognitive status can be attributed to their ability to use the GBA communication systems to positively impact both the brain and the gut (O’Callaghan and van Sinderen, 2016). As a result, the use of dietary therapies to influence changes in neurological disorders is a compelling and imperative field of research.

## The effects of food on the gut–brain axis

Although associations between diet, psychosocial stress, and neurodegenerative diseases have been reported, determining causal relationships in human studies is difficult. Nonetheless, numerous studies on AD models in recent years have supported nutritional therapies and dietary interventions, especially when subjects adhere to “AD-protective” diets, such as the Mediterranean-DASH Intervention for Neurodegenerative Delay (MIND) diet, which is a hybrid of the Mediterranean and Dietary Approaches to Stop Hypertension (DASH) diets (van den Brink et al., 2019). Conversely, Western dietary patterns, which typically consist of processed foods, convenience products, snacks, and sugary soft drinks, are deficient in essential nutrients, such as fibers, vitamins, and minerals, that are essential for preventing chronic metabolic inflammation (Christ et al., 2019). Several studies have discovered that such dietary patterns consisting of large amounts of processed carbohydrates, saturated fats, and so-called “empty” calories (Gush et al., 2021) exacerbate the inflammatory conditions associated with AD.

Another issue of interest is the role of probiotics and fermented foods in the prevention of AD through bioactive metabolites, an overall process that may be achievable through neuroendocrine/neuroactive compounds, gut microbiota diversification, and other neurological effects (Xiang et al., 2022). Though nutritional therapies have not been established as a preemptive measure for preventing or reversing AD, various studies suggest that future research should be conducted to explore the pathophysiological process and the subsequent development of efficacious, nonpharmacological strategies for AD prevention.

### Probiotics and fermented foods

Probiotics are live microorganisms that provide a health benefit to the host by supporting a healthy digestive tract when applied to the body (Hill C. et al., 2014). Fermented foods, which are defined as “foods made through desired microbial growth and enzymatic conversions of food components,” are related to this process (Marco et al., 2021). Various fermented foods, most notably kimchi, yogurt, kefir, kombucha, sourdough bread, and soybean products, contain live cultures necessary for fermentation that have the potential to provide probiotic functions by modulating the gut microbiota, though human clinical studies are limited (Mota de Carvalho et al., 2018).

Common probiotic strains found in fermented foods include yeasts and a group of Gram-positive, non-spore-forming bacteria known as lactic acid bacteria (LAB) (Mokoena, 2017), typically consisting of *Lactobacillus* (Heller, 2001), *Streptococcus*, *Leuconostoc*, and *Bifidobacterium* (Rezac et al., 2018). Probiotic functions of LAB include inhibiting pathogenic bacteria colonization through competition and antimicrobial compounds, regulating intestinal transit, enhancing the intestinal barrier through anti-inflammatory cytokines and intestinal/epithelial cell growth factors, and regulating immune responses (Wang J. et al., 2018).

Probiotics also support the host by enhancing the production of nutritive compounds, such as antioxidant enzymes (Wang et al., 2017b), vitamin C, phenolic compounds, conjugated linoleic acid, folate (B9), and carotenoids (Peng et al., 2020; Xu Y. et al., 2020a; Pourbaba et al., 2021). LAB, *Bifidobacterium*, and other commensal bacteria provide additional vitamins, including riboflavin, vitamin 12, and vitamin K, which are essential to body functions, as well as SCFAs through carbohydrate fermentation (LeBlanc et al., 2017). Neuroactive compounds produced by probiotics may be significant to cognitive health, including serotonin, GABA, histamine, arachidonic acid, and adrenaline (Ma et al., 2020). Thus, certain fermented foods and probiotics may play crucial roles in neuroprotection through their interactions with the immune, endocrine, and nervous systems, the intestinal barrier, and the BBB.

#### The effect of probiotics on the gut–brain axis

One overarching theme linking specific probiotics to the prevention of neurodegeneration is potent anti-inflammatory action through the microbial modulation of antigen-presenting innate immune cells (macrophages, dendritic cells, and B-cells) in the subepithelial lamina propria tissue (Mann and Li, 2014) which positions the immune system in close proximity to the gut microbiota, antigens, and any pathogens that cross the epithelial barrier, allowing Toll-like receptors (TLRs) on these immune cells to recognize microbe-associated molecular patterns (MAMPs) and pathogenic ligands that initiate signaling cascades (Hug et al., 2018). Additionally, commensal microbiota produces a variety of secondary neuroactive molecules that interact with molecular signaling cascades (Morgan et al., 2007; Thomas et al., 2012; Tomaro-Duchesneau et al., 2012; Gim et al., 2013), which all play an extensive role in endocrine, lipogenesis, and apoptosis mechanisms that co-regulate key CNS processes with the brain (Clarke et al., 2014).

##### Neurotransmitter precursors

Some probiotics encode genes for specific enzymes that convert dietary substrates into corresponding neurotransmitters or precursors (Serrano-Pozo et al., 2011) while others act as signaling molecules to induce this process and stimulate enteroendocrine cells (Yano et al., 2015; Ceppa et al., 2020). Generally, neurotransmitters (e.g., glutamate, dopamine, serotonin, GABA) must be synthesized in the brain from local pools of amino acids and acted upon by corresponding neurotransmitter-producing cells and intermediate host enzymes, as functional neurotransmitters themselves cannot penetrate the BBB (Zaragozá, 2020; Chem et al., 2021). For example, enterochromaffin cells take up tryptophan from dietary protein to synthesize serotonin, a process that is regulated by the bacterial kynurenine synthesis pathway (Bailey and Cryan, 2017; de et al., 2018), where spore-forming probiotics (predominantly *Clostridia*) can promote the biosynthesis of serotonin and decarboxylation of the precursor 5-hydroxytryptophan (Yano et al., 2015; Luqman et al., 2018). Furthermore, colonic 5-HT production is influenced by various other probiotic metabolites (SCFAs) by promoting stimulatory activities on enterochromaffin cells (Reigstad et al., 2015; Yano et al., 2015). Accordingly, the dietary origins of these precursors enable the modified intestinal microbiota to influence host behavior by regulating the metabolism of these neurotransmitter precursors.

#### In vivo studies

##### Lactobacillus

Extensive studies in recent years have tried to establish the connection between Gram-positive bacteria, especially from the order *Latobacillales*, and the prevention of microglia-mediated neuroinflammation (Saez-Lara et al., 2015; Morshedi et al., 2019). A study on the spatial memory of rats with Aβ1–40 intrahippocampal injection used a probiotic mixture of *Lactobacillus reuteri*, *Lactobacillus rhamnosus*, and *Bifidobacterium infantis* to demonstrate decreased Aβ aggregation in the hippocampus, augmented antioxidant enzymatic activity, reduced oxidative stress markers, reduced levels of inflammation (namely IL-1β, IL-6, and TNF-α), and an increase in anti-inflammatory responses (IL-10) (Mehrabadi and Sadr, 2020). A follow-up study that compared blood and stool specimens of AD patients discovered that using a probiotic treatment significantly decreased IL-6 levels and increased IL-10 levels (Mombelli et al., 2020). A different study comparing similar serum and stool specimens of AD patients after a similar probiotic treatment of *Lactobacillus*, *Lactococcus*, and *Bifidobacterium* strains reported a protective adjustment to inflammation as well as an increase in kynurenine serum levels and tryptophan breakdown (Leblhuber et al., 2018).

Additionally, the gut status of a Drosophila melanogaster AD model before and after *Lactobacillus plantarum* DR7 administration revealed an overall reduction in Wolbachia and an increase in *Acetobacter* populations (Tan et al., 2020) while Abraham et al. (2019) determined that the supplementation of *Lactobacillus acidophilus*, *Bifidobacterium longum*, and various vitamins increased butyrogenesis and spatial memory, suggesting that gut dysbiosis accompanying AD can be reversed. While its role in cognitive function and the progression of AD has not been elucidated, bacteria from the *Latobacillales* order exhibit immunomodulatory and gut-modulatory effects.

##### Bifidobacterium

*Bifidobacterium* is another probiotic that has been identified as a beneficial modulator of the GBA by maintaining bodily functions and deterring pathogens (Unno et al., 2003). A study aimed at investigating the prevalence of AD pathophysiology after Bifidobacterium Lactis Probio-M8 intake quantified the number and size of Aβ plaques in APP/PS1 mice, and the researchers concluded that the specific probiotic treatment attenuated cognitive impairment by restoring the richness of a gut microbiota lacking in *Lactobacillus* and *Streptococcus*, resembling the gut diversity of a pre-treatment younger status (Cao et al., 2021). Interestingly, Kobayashi et al. (2017) discovered another strain of *Bifidobacterium* that did not affect the gut microbiota of AD model mice to a great extent at phylum levels but significantly increased the population of *Actinobacteria* and family *Bifidobacteriaceae* and may have even suppressed Aβ-induced changes by modulating genes associated with immune response in the hippocampus. The use of probiotics may be a strategy to promote Aβ clearance by regulating immune-reactive genes induced by Aβ (Tang et al., 2021) and not exclusively through the suppression of proinflammatory biomarkers.

##### Other probiotic strains

Other intestinal commensal bacteria with probiotic characteristics include *Bacillales* strains from the Firmicutes phylum and certain *Clostridium* clusters, although there are risks associated with some *Clostridium* species due to exotoxin release (Guo et al., 2020). A study reported that β-cell mass, insulin resistance, and A*beta* deposition were normalized in AD rats fed a diet consisting of probiotic-infused chungkookjang (fermented soybeans), indicating that *Bacillus licheniformis* stimulated key pathways in glucose metabolism (Yang et al., 2015). Resistance to brain insulin signaling and impaired glucose metabolism are common features of AD, obesity, and diabetes, implying the existence of common mechanisms underlying these disorders (Ferreira et al., 2018). The decreased levels of insulin-like growth factors-I and –II displayed in AD brains accompanied by the attenuation of insulin-phosphoinositide 3-kinase-Akt signaling activates GSK-3b (a major kinase that increases tau phosphorylation) and decreases glucose and Aβ uptake by astrocytes (Yang et al., 2015; Ferreira et al., 2018). Similarly, Sun et al. (2020) discovered that *Clostridium butyricum* treatment ameliorated cognitive deficits and decreased Aβ oligomerization in APP/PS1 mice by reversing abnormal butyrate levels and gut microbiota alterations, which helped to suppress microglial activation. The comparative effects of probiotic formulations are further characterized in Table 1.

**TABLE 1:** Summaries of documented effects of probiotic formulations on AD models through the GBA.

Probiotics	Subject/Model	Effect	References
*Lactobacillus reuteri*, *Lactobacillus rhamnosus*, *Bifidobacterium infantis*	Aβ1–40 injected Wistar rats	Decreased Aβ aggregation in the hippocampus and augmented antioxidant enzymatic (SOD) activity. Reduced levels of IL-1β, IL-6, and TNF-α while increasing levels of IL-10	Mehrabadi and Sadr, 2020
*Lactobacillus casei W56, Lactococcus lactis W19, Lactobacillus acidophilus W22, Bifidobacterium lactis W52, Lactobacillus paracasei W20, Lactobacillus plantarum W62, Bifidobacterium lactis W51, Bifidobacterium bifidum W23, Lactobacillus salivarius W24.*	Human AD patients	Increased kynurenine serum levels and modulated the tryptophan pathway involving indoleamine 2,3-dioxyngease-1. Increased BDNF expression and synapsin in the hippocampus	Leblhuber et al., 2018
*Lactobacillus plantarum* DR7	*Drosophila melanogaster*	Restored gut microbiota diversity in AD through the reduction in Wolbachia and increases in *Acetobacter* and *Stenotrophomonas* populations	Tan et al., 2020
*Lactobacillus acidophilus*, *Bifidobacterium longum*	APP/PS1 mice	Increased *B. thetaiotaomicron* and *L. johnsonii* levels in the gut microbiota. Increased butyrogenesis and decreased number of Aβ plaques, improving performance on The Morris Maze Test for spatial learning	Abraham et al., 2019
Bifidobacterium Lactis Probio-M8	APP/PS1 mice	Reduced levels of *distasonis*, *Streptococcus, Adlercreutzia*,and *Lactobacillus* populations while increasing levels of *Desulfovibrionaceae, Oscillospira, Coprococcus, Clostridiales*, and *acidifaciens.* Upregulation of tryptophan metabolism and a reduction of Aβ plaques in the hippocampus helped improve spatial working memory through the Y-maze test	Cao et al., 2021
Bifidobacterium breve strain A1	Aβ25–35 and Aβ1–42 injected mice	Increased Actinobacteria and Bifdobacteriaceae populations. Significantly increased plasma concentration of acetate and BDNF upregulation. Was found to modulate genes associated with immune response in hippocampus, exhibiting ameliorative effects on cognitive dysfunction in both working memory and long-term memory	Kobayashi et al., 2017
*Bacillus licheniformis*	Aβ25–35 injected male Sprague–Dawley rats	Stimulated key glucose metabolism pathways and hippocampal insulin signaling (pAkt → pGSK → pTau) to normalize β-cell mass, insulin resistance, and Aβ deposition.	Yang et al., 2015
*Clostridium butyricum*	APP/PS1 transgenic mice	Decreased Aβ oligomerization, reversed abnormal butyrate levels, and restored gut microbiota alterations. Supplementation helped suppress chronic microglial activation, reduced Aβ deposits, prevented production of TNF-α and IL-1β, and suppressed phosphorylation of NF-κB p65	Sun et al., 2020
*Lactobacillus acidophilus*, *Lactobacillus casei, Lactobacillus fermentum*, *Bifidobacterium bifidum*	Human AD patients	Decreased the plasma malondialdehyde and serum high-sensitivity C-reactive protein levels. Insulin resistance and β cell function were improved. Twelve-week intervention resulted in an improvement in Mini-Mental State Examination (MMSE) score	Akbari et al., 2016
*Eubacterium rectale*	BD (Behçet’s disease) mice	Reduced the frequency of the dendritic cell activation marker CD83+ and significantly increased the frequency of NK1.1+ cells, improving symptoms of systemic inflammation	Islam et al., 2021
*Lactobacillus plantarum*	*L. plantarum* ZLP001-treated piglets and normal human subjects	Increased the abundance of butyrate-producing bacteria *Lactobacillus, Anaerotruncus*, and *Faecalibacterium* while decreasing inflammatory *Clostridium sensu stricto* 1. Significantly counteracted the increase in gut permeability induced by enterotoxigenic *E. coli.* Alleviated the reduction in tight junction proteins and downregulated IL-6, IL-8, and TNFα. Supplementation enhanced the diversity of microbial neuroactive metabolites	Wang J. et al., 2018; Ma et al., 2020
*Lactobacillus casei + myosin cross-reactive antigen*	BALB/cJ mice	Stimulates the conversion of conjugated linoleic acids. Competitively reduced the infection with *Salmonella enterica* serovar Typhimurium and enterohemorrhagic *E. coli*. Conferred better protection against negative hematological changes, intestinal histological alterations, and pathogen-induced gut inflammation	Peng et al., 2020
*L. rhamnosus and L. casei, L. paracasei, L. plantarum, L. fermentum, L. reuteri, L. sakei*	Juvenile male F344 rats	Significantly increased BDNF expression and markers for neuronal plasticity (mRNA expression for cfos and the NMDA receptor). Positively correlated with altered gene expression for serotonin receptors within multiple brain regions. Impacted anxiety-related behavior and underlying emotion regulation	Mika et al., 2018
*Bifidobacterium longum* subsp. *Longum JCM 1217*	Germ-free BALB/c mice	Increased production of acetate and genes encoding an ATP-binding-cassette-type carbohydrate transporter provided protection from *E. coli* O157:H7 by improving intestinal defense. The translocation of toxins from the gut lumen to the blood was inhibited.	Fukuda et al., 2011

Aβ, amyloid beta; APP/PS1, amyloid precursor protein/presenilin 1; IL-1β, interleukin-1β; IL-6, interleukin-6; IL-10, interleukin-10; TNF-α, tumor necrosis factor-α; BDNF, brain-derived neurotrophic factor; pAkt, phosphorylated protein kinase B; pGSK, phosphorylated glycogen synthase kinase 3; pTau, phosphorylated tau; NF-κB, Nuclear factor-κB; CD, cluster of differentiation 83; NK, natural killer; BD, Behçet’s disease; NMDA, N-methyl-D-aspartate.

#### Conflicting evidence

Although there is substantial evidence for the abilities of probiotics and enhanced gut diversity to address gut inflammation and delay neurodegeneration, several other studies do not report such an anti-inflammatory effect on the brain, or at least a significant one. Agahi et al. (2018) reported that neither the cognitive function nor antioxidant biomarkers in AD patients were affected by probiotic supplementation consisting of *Lactobacillus acidophilus*, *Lactobacillus casei, Lactobacillus fermentum*, and *Bifidobacterium bifidum*, Similarly, a meta-analysis concluded that there were no forms of probiotic/prebiotic intervention that had a significant effect on global or specific domains of cognition, including fermented foods (Marx et al., 2020).

However, common reasons for these inconsistencies include the disease severity or stage, varying formulations of probiotic bacteria, supplement exposure time, the use of different cognitive-related inflammatory biomarkers and cognitive test designs, heterogeneity regarding the populations and sizes used, and an overall limited number of statistically powered studies (Agahi et al., 2018; Marx et al., 2020). Differences in the severity of the disease stage, as well as duration and timing of supplement administration, may be particularly crucial, as the loss of synapses and NFT development are irreversible pathological changes in later stages of AD and act as general thresholds of irreversible neurotoxicity (contributing to the failure of many anti-Aβ drugs) (Husna Ibrahim et al., 2020). In fact, Akbari et al. (2016) and Marx et al. (2020) conducted another clinical trial on people with multiple sclerosis using the same probiotic supplementation and observed a positive effect on motor behavior and gene expression of biochemical factors, indicating that the probiotics initially used have modulatory capabilities and that different factors specific to AD are negating the effects of probiotic interventions.

Thus, inconsistent effects of supplements on neurodegeneration should not be construed as paradoxical results, as factors, including age and stage of the disease, must still be considered (Agahi et al., 2018). Future studies are required to identify the most effective probiotic strains in the inhibition of AD progression, as well as the necessary timing for such therapies within a complex neurotoxic cascade.

### Mediterranean and Mediterranean-DASH intervention for neurodegenerative delay diets

The Mediterranean diet pattern is renowned for being one of the healthiest diets in the world, consisting of vegetables, fruits, whole grain, legumes, nuts, vegetable oils, fish, shellfish, white meat, eggs, and dairy products, which provides a healthy profile of dietary fiber, low glycemic index, anti-inflammatory effects, and various antioxidants (Castro-Quezada et al., 2014). The Mediterranean-style diet (MeDi) lowers the risk of deficiencies in micronutrient intake and balances the intake of certain macronutrients, as it supplies high amounts of B group vitamins, antioxidant vitamins (vitamins E and C), and carotenes while increasing monounsaturated fatty acid (MUFA) consumption (Castro-Quezada et al., 2014; Akbari et al., 2016).

The Mediterranean diet and associated lifestyle habits can improve cardiovascular (Martínez-González et al., 2019) and cognitive health (Karstens et al., 2019) by providing better qualities of dietary fat, bioactive compounds, and overall nutrition, prompting the organization of health promotion strategies incorporating this diet, especially in populations with significant micronutrient deficiencies.

#### Dietary fiber

Dietary fibers are carbohydrates that are not hydrolyzed by the endogenous enzymes in the small intestine of humans (Berding et al., 2021). Prebiotic oligosaccharide isolates, or dietary carbohydrates that are selectively fermented by gut microbiota to confer health benefits to the host, are a well-known type of dietary fiber that reach the site of action in the colon and are fermented by saccharolytic microbes, such as *Bifidobacteria* (Kelly et al., 2021). To note, while several murine studies have demonstrated the psychophysiological effects of prebiotics, there are very few studies that have examined the targeting of the GBA in humans (Peterson, 2020).

The metabolization of various dietary fibers with complex chemical structures, such as starch or cellulose (Dhingra et al., 2012), has been associated with an increase in microbial diversity ranging in strain specificities and enzymatic capacities (Flint et al., 2008; Martínez et al., 2010; Alfa et al., 2018) that usually highlight several beneficial microbes, such as *Bifidobacteria*, *Lactobacilli, Ruminococcus, and Akkermansia muciniphila* (Berding et al., 2021), as well as the inhibition of pathogens (e.g. some *Clostridium* species, *Enterococcus*, *Escherichia* (So et al., 2018; Tangestani et al., 2020)). Independently of the gut microbiota, dietary fiber can interact locally with enterocytes, dendritic cells, macrophages, and monocytes and support epithelial barrier function by promoting the assembly of tight junction proteins or intestinal epithelial cell proliferation through AMP-activated protein kinase (AMPK), epidermal growth factor receptors, or TLR mechanisms (Bindels et al., 2017; Cai et al., 2020). Besides local effects, the growth of beneficial gut commensals and their metabolites associated with high fiber intake could be more relevant to the more distant brain-modulating properties.

##### The role of short-chain fatty acids in the gut–brain axis

SCFAs, the most obvious route of communication between the gut and brain that is mediated by dietary fibers, are absorbed into the portal circulation and mediate neurological signals principally *via* interactions with orphan G protein-coupled receptors/free fatty acid receptors and the inhibition of histone deacetylases (HDACs) (Offermanns, 2014; Koh et al., 2016). Through free fatty acid receptors, propionate initiates gluconeogenesis in the gut lumen through afferent circuits (Grompone et al., 2012), such as the dorsal motor nucleus of the vagus nerve (Lacassagne and Kessler, 2000), which accentuates central signaling processes (Sjögren et al., 2002). Importantly, the vagus nerve can be activated by fiber-responsive bacteria such as *L. rhamnosus* to stimulate BDNF expression (Berding et al., 2021).

Apart from direct vagal stimulation, SCFAs can act as endocrine signaling molecules that can easily be transported across the BBB by monocarboxylate transporters (Morgan and Zheng-gang, 2010) to influence brain biochemistry and longevity such as the Foxo gene locus (Braniste et al., 2014; Pino et al., 2014; Vidovic et al., 2018) by the HDAC inhibitory activity of butyrate (Chriett et al., 2019). Dietary fibers can mediate the production of glucagon-peptide 1, PYY, and ghrelin, gut-derived hormones that can cross the BBB and reach the brain, as such messengers can be stimulated by both gut microbes and SCFAs (Bauer et al., 2016).

Additionally, SCFAs modulate the expression of several neurotransmitter mediators, including the production of catecholamines through tyrosine hydroxylase expression (Fukumoto et al., 2003), stimulation of serotonin receptors on the vagal sensory fibers (Shah et al., 2006), and GABA receptors (Nankova et al., 2014). Although functional neurotransmitters cannot directly be produced from dietary fiber, bacterial species such as *Lactobacillus*, which are known neurotransmitter producers, respond to dietary fiber (Kato-Kataoka et al., 2016; Mika et al., 2018). Besides protecting the BBB against neurotoxic factors, SCFAs and consequently dietary fibers modulate the kynurenine pathway, which can cross the BBB and metabolize into kynurenic acid (Westfall et al., 2017). Thus, it is certain that there are many underlying avenues of the fiber-brain crosstalk that have been identified and will surely emerge.

#### Fatty acids

##### Monounsaturated fatty acids

MUFAs include one double bond in the fatty acid chain with the remainder single-bonded (Hammad et al., 2016). The alleviation of endothelial dysfunction and reduced insulin concentrations by the consumption of the MeDi may be linked to high consumption of MUFAs such as oleic acid that are derived from extra virgin olive oil (Ye et al., 2021), which enhances the gut microbiota diversity of models under risk of metabolic syndrome (Pu et al., 2016; Hidalgo et al., 2018; Lang et al., 2018) by raising the Firmicutes: Bacteroidetes ratio, *Bifidobacterium* (Actinobacteria) populations in humans (Machate et al., 2020), and *Bifidobacteria* to *E. coli* ratios (Miller C.B. et al., 2021). Indeed, further studies suggest that replacing SFA-enriched diets with MUFA such as the MeDi had a positive impact on butyrate-producing bacterial populations, notably the *Bifdobacteriaceae* family, *Prevotella, Turicibacter*, *Roseburia*, *Oscillospira*, and *Verrucomicrobia* (Wolters et al., 2019).

Thus, MUFAs may alter gut microbiota structure to favor SCFA-producing organisms, and an anti-inflammatory profile has been described (Michielsen et al., 2019). Studies comparing mice raised on a MUFA-rich diet versus a SFA-rich diet show higher circulating levels of anti-inflammatory markers, including IL-4, IL-10, and PPARγ, as well as lower levels of pro-inflammatory markers such as IL-6, IL-8, MCP-1, IL-1β, CRP, and TNF-α (Tamer et al., 2020). In correlation, MUFAs decrease M1 macrophage infiltration and stimulates M2 macrophage polarization (Ravaut et al., 2021), especially through adiponectin expression (Lovren et al., 2010) and the treatment of immune cells with palmitoleate (Chan et al., 2015). Palmitoleate decreases NF-κB nuclear translocation via the stimulation of PPARγ and the phosphorylation of AMPK, which increases MGL2, IL-10, TGFβ1, and MRC1 (Yang et al., 2010; Chan et al., 2015). Another prominent MUFA is oleate, which inhibits LPS-induced IL-1β maturation (Finucane et al., 2015), reverses cell death pathways activated by SFAs (Sieber et al., 2010), and protects various cells from palmitic-mediated insulin resistance (Coll et al., 2008). Though there is a lack of substantial studies so far, the addition of MUFA in diets may be a nutraceutical avenue to ameliorate the general metabolic profile of AD.

##### Polyunsaturated fatty acids

Omega-3 (n-3) long-chain polyunsaturated fatty acids (n3 LC-PUFA), which are also highlighted in the MeDi, have demonstrated the ability to increase SCFA-producing organisms, such as *Bifidobacterium* and *Lactobacillus* (Ghosh et al., 2013), and suppress mediators of mucosal inflammation such as *Helicobacter*, *Enterobacteria*, and Firmicutes (Yu et al., 2014), which reduces impaired cytokine/chemokine induction (Ghosh et al., 2013), suppresses LPS-producing bacteria to address endotoxemia conditions (Kaliannan et al., 2015), and enhancing intestinal wall integrity (Costantini et al., 2017). Furthermore, n3 LC-PUFAs could modulate intestinal epithelial barrier integrity and overall enteral health through the regulation of intestinal alkaline phosphatase to modify intestinal membrane pH, as proven by eicosapentaenoic acid (EPA), docosahexaenoic acid (DHA), and α-Linolenic acid (Costantini et al., 2017). These essential fatty acids could attenuate the decrease of the Firmicutes: Bacteroides ratio (Liu et al., 2012), detoxify LPS (Kaliannan et al., 2015), and be incorporated into microbiota membranes, which modifies adherence to the intestinal membrane (Ye et al., 2021). The resolution of inflammation has also been observed, where DHA could inhibit TLR4 activation and address LPS levels (Hwang et al., 2016) while EPA could increase TGF-β synthesis (Liu et al., 2012). Of interest is the n3/n6 PUGAs ratio, which may be implicated in the promotion of anti-inflammatory effects (Simopoulos, 2016) and the modulation of endocannabinoid signaling, which is involved in brain development, cytokine release from microglia, neurotransmitter release, and synaptic plasticity (Cristino et al., 2020).

#### Antioxidants

##### Vitamins

The nutrients having antioxidant potential can both stop the excess production of free radicals and promote scavenging of prevailing free radicals in the host body (Eke et al., 2017), which supports cellular and humoral immune responses (Crump et al., 2013). Vitamins are associated with various microbial synthesis pathways, being produced by and influencing the gut microbiota composition (Sun, 2010) and reducing inflammatory markers such as IL-6 and TNF-α (Shirpoor et al., 2016). In addition, vitamin E such as α-tocopherol and γ-tocopherol-rich tocopherols seem to be implicated in the mitigation of mucosal tissue damage *via* altering the composition of gut microbiota in colitis-induced mice (Liu et al., 2021), as well as exhibiting powerful antioxidant properties on lipoproteins and cell membranes (Hasegawa et al., 2001). Moreover, vitamin E was associated with the capacity to modulate the Firmicutes: Bacteroidetes ratio at the phylum level (Yang et al., 2020), lower inflammatory mediators from the Proteobacteria and Firmicutes phylum (Mandal et al., 2016), and increase butyrate-producing bacteria, such as *Roseburia* (Tang et al., 2016). The redox state of vitamin C, another essential antioxidant, was also found to restore *Lactobacillus*, *Bifidobacterium*, and *Coriobacteriaceae* populations as well as decreasing Bacteroidetes and *E. coli* (Xu et al., 2014). Furthermore, vitamin C impacts the chemotaxis process in neutrophils and the phagocytosis process of microbes by protecting cells from oxidative explosions (Cerullo et al., 2020).

##### Polyphenols

Polyphenols sequester the ROS and RNS to prevent the formation of toxic Aβ oligomers and NFTs (Ruotolo et al., 2020). Polyphenols that can selectively detoxify Aβ oligomers are generally capable of crossing the BBB (Hwang and Yen, 2008) such as hesperetin, hesperidin, neohersperidin, citrus flavanones, and various aryl-γ-valerolactone and arylvaleric acid derivatives, which are major compounds of derived from the bacterial metabolization of flavan-3-ols (Unno et al., 2003; Reddy et al., 2020). Further metabolism of the valerolactones results in the formation of bioavailable phenolic degradation products and secondary polyphenolic metabolites, which have demonstrated greater BBB permeability *in vivo* and can attenuate neuroinflammation (Carregosa et al., 2020). Additionally, dietary polyphenolic compounds undergo extensive catabolism in the colon to form small polyphenolic compounds such as urolithins and pyrogallol, which are effective as antioxidant agents (Verzelloni et al., 2011) and can attenuate the neuroinflammation in BV2 microglia through the NF-κB, MAPK, and Akt signaling pathways (Xu et al., 2018). Indeed, Taxifolin, a naturally occurring flavonoid polyphenolic compound, exhibited neuroprotective effects in mice models by reducing the production of TREM2 (Reddy et al., 2020).

##### Carotenoids

Besides exerting various antioxidant properties through the neutralization of ROS, several carotenoids have showcased anti-neuroinflammatory properties that address LPS-induced mechanisms in microglia, such as lutein through the inhibition of NF-κB and lipid peroxidation (Kim et al., 2008; Wu et al., 2015) and Fucoxanthin through the inhibition of NF-κB, protein kinase B, and MAPK signaling and subsequently TNF-α, ROS, IL-6 production (Lee et al., 2021). Crocetin and crocin were found to inhibit LPS-induced nitric oxide and cytokine formation in microglia and block the effects of LPS on hippocampus cell death (Nam et al., 2010), while Ilycopene decreased LPS-induced expression of IL-1β, heme oxygenase-1, IL-6, and TNF-α throughout the plasma and brain in murine models (Sachdeva and Chopra, 2015; Zhang et al., 2016). Collectively, such findings suggest that carotenoids act as strong anti-inflammatory agents in the CNS. Additionally, β-carotene is a precursor to vitamin A, which could increase the abundance of Lactobacillus to protect against norovirus infections (Rackerby et al., 2020) and increase other bacteria, such as *Allobaculum*, *Akkermansia*, and *Bifidobacterium* (Lee and Ko, 2016), promoting intestinal barrier function. The restorative function of vitamin A on the Firmicutes: Bacteroidetes ratio is also an interesting area of study (Nan et al., 2021).

#### Clinical studies

##### Mediterranean diet

Studies on the MeDi form a clearer consensus regarding the relationship between human gut modulation and cognitive performance compared to the exclusive use of probiotics and/or AD models. A study consisting of middle-aged adults discovered that lower MeDi adherence correlated with higher rates of Aβ deposition, hypometabolism, hypertension, and a greater number of AD biomarker abnormalities (e.g., higher C-Pittsburgh compound B PET deposition), while higher MeDi adherence was estimated to provide up to 3.5 years of protection against AD due to the normalization of the aforementioned observations (Berti et al., 2018) and could potentially alleviate prodromal AD symptoms (Ballarini et al., 2021). Notably, the MeDi has higher scores of monounsaturated/saturated fat ratios, whose phenolic components have been associated with reduced AD pathology in animal models (Qosa et al., 2015; Román et al., 2019) and a lower risk of mild cognitive impairment (Martinez-Lapiscina et al., 2013; Ballarini et al., 2021; Tzekaki et al., 2021).

Additionally, a modified Mediterranean-ketogenic diet is thought to stimulate intestinal SCFA production and reduce the abundance of genetic pathways associated with bacterial toxins, energy balance, and inflammation (Nagpal et al., 2019; Rusek et al., 2019). Due to the production of ketone bodies through such diets and their ability to reverse energy hypometabolism, regulate glutamate release, and promote Aβ clearance across the BBB (Versele et al., 2020), Mediterranean diets enriched with isocaloric coconut oil are considered an alternative to glucose for brain metabolism and sustainment of neurogenesis through the assembly of a larger number of medium-chain triglycerides (de la Rubia Ortí et al., 2018; Chatterjee et al., 2020).

##### Mediterranean-DASH intervention for neurodegenerative delay diet

The concept of a combination of modified diets has been extended to Mediterranean and DASH diets, producing a hybrid form known as the MIND diet (Castro-Quezada et al., 2014). Multiple cross-sectional, longitudinal, and intervention studies on the elderly with an increased risk for AD support the mitigation of cognitive deficits in AD based on a higher MIND diet adherence score (Castro-Quezada et al., 2014; Tangney et al., 2014; Morris et al., 2015; Mosconi et al., 2018; van den Brink et al., 2019; Wesselman et al., 2021). These strong associations between dietary patterns and cognitive domains may have been mediated by effects on medial temporal atrophy and cerebral amyloid pathology through the gut microbiota and immune system modulation (Ballarini et al., 2021; Wiȩckowska-Gacek et al., 2021). In some cases, the recently developed MIND diet was discovered to be more effective compared to the independent use of either the Mediterranean or DASH diets, revealing a stronger inverse association with AD pathogenesis (Castro-Quezada et al., 2014; Hosking et al., 2019) and premature death in the highest tertile of adherence (Corley, 2020). The superior effectiveness of the MIND diet in recent studies may be explained by the consumption of nuts, the synergistic action of additional dietary components specific to neuroprotection excluded in the MeDi (including unique categories of green leafy vegetables and berries providing potent polyphenols), as well as the restriction of particular less-healthy foods used in the calculation of the MIND score and not usually accounted for in the MeDi (Hosking et al., 2019; Corley, 2020). Longitudinal and cross-sectional studies investigating the association between adherence to the MeDi/MIND diet and cognitive health are outlined in Table 2.

**TABLE 2:** Results of included studies investigating the relationship between MeDi/MIND diet adherence and cognitive performance.

Study	Population	Duration	Intervention	Results
Ballarini et al. (2021)	Cognitively normal participants and individuals at higher AD risk from the German multicenter DELCODE (mean age 69.5 ± 5.9 years)	Cross-sectional study	Participants were administered the German adaption of the semiquantitative European Prospective Investigation into Cancer FFQ. For alcohol, a moderate consumption (10–50 g/d in men and 5–25 g/d in women) was considered beneficial. Employed 0–9 point MeDi score	Higher MeDi adherence related to larger mediotemporal gray matter, favorably moderated the Aβ42/40 ratio, and decreased pTau181 pathology. A 1-point increase in MeDi score corresponded to –0.84 years of age
Martinez-Lapiscina et al. (2013)	Cognitively normal participants at high CVD risk from the PREDIMED-NAVARRA trial (mean age 74.1 ± 5.7 years)	6.5 years	Participants were administered a 137-item FFQ and received intensive education to increase adherence to MeDi diet. Participants were divided into two groups, receiving either EVOO (1 L/week) or 30 g/day of raw, unprocessed mixed nuts	Significantly better performance across fluency and memory tasks were observed for participants in the MeDi+EVOO group in comparison to a low-fat control diet
Berti et al. (2018)	Cognitively normal participants from the New York University School of Medicine/Weill Cornell Medicine College (mean age 50 ± 8 years)	3 years	Participants were administered the Harvard FFQ. Food items were combined into 30 food groups based on composition. Employed 0–9 point MeDi score	Higher MeDi adherence increased FDG-PET glucose metabolism (CMRglc) and reduced PiB-PET deposition in AD-affected regions. Higher adherence was estimated to provide 1.5–3.5 years of protection against AD
Morris et al. (2015)	Cognitively normal participants from the Rush Memory and Aging Project (mean age 81.4 ± 7.2 years)	4.7 years	Participants were administered a modified 144-item Harvard semi-quantitative FFQ. Employed 0–15 point MIND diet score	The MIND score was positively associated with slower decline in the five cognitive domains and global cognitive score. The difference in decline rates between the highest and lowest tertile of diet scores was the equivalent to being 7.5 years younger in age
Wesselman et al. (2021)	Cognitively normal participants and patients with MCI from the DELCODE interim baseline data release (mean age 69.3 ± 5.6 years)	Cross-sectional study	Nutritional intake was assessed using the German version of the 148-item European Prospective Investigation into Cancer FFQ. Principle component analysis was used to identify data-derived dietary patterns based on 39 food groups. Employed both 0–9 point MeDi score and 0–15 point MIND diet score.	Adherence to the MeDi and MIND diet was associated with better memory. The ‘alcoholic beverages’ PCA component was positively correlated with better language, executive functioning, and working memory. Exclusion of MCI subjects revealed a positive impact on language functions.
Mosconi et al. (2018)	Cognitively normal participants affiliated with the New York University School of Medicine (mean age 50 ± 6 years)	Cross-sectional study	Participants were administered the Block or Harvard FFQ, the Baecke and Minnesota leisure time physical activity questionnaires, and a 25-item intellectual activity interview. Employed 1–8 point MeDi score.	Adherence to the MeDi and insulin sensitivity were positively associated with MRI-based cortical thickness. Intellectual enrichment and increased BMI explained variability in cognitive performance
Hosking et al. (2019)	Cognitively normal participants from the 1960 cohort of the Personality and Total Health Through Life study (age 60–64 years)	12 years	Participants were administered the 183-item Commonwealth Scientific and Industrial Research Organization semi-quantitative FFQ. Employed 0–15 MIND diet score, including the 0–9 point MeDi score and the 0–55 point traditional Greek MeDi score	For every 1-point increase in MIND diet score, the odds of MCI/cognitive impairment decreased by 19%. There were no associations between the 9-point MeDi score and incidence of dementia. The highest tertile of MIND diet consumption was associated with a 53% reduction in the odds of impairment compared with lower levels of intake
Corley (2020)	Cognitively normal participants from the Lothian Birth Cohort 1936 Study (mean age 69.5 ± 0.8 years)	12 years	Dietary intake was assessed at baseline using a semi-quantitative 168-item Scottish Collaborative Group FFQ, which excluded questions for butter/margarine consumption. Factor scores for the MeDi were calculated individually by summing the intakes of food groups weighted by their factor loadings. Employed 0–14 point MIND diet score.	The MIND and MeDi were associated with a lower mortality risk. The risk of death was reduced by 12% per unit increase in MIND diet score. The top tertile of MIND diet scores had a 37% lower risk of death overall compared to the lower tertile.
Tangney et al. (2014)	Cognitively normal participants from the Memory and Aging Project (mean age 81.5 ± 7.1 years)	4.1 years	Participants were administered the modified 144-item version of the Chicago Health and Aging Project semiquantitative FFQ. Food items were computed using the Harvard nutrient database and assigned to 7 DASH food groups. Employed both 0–10 point DASH diet score and 0–9 MeDi score	A 1-unit change in the DASH and MeDi scores were associated with slower rates of global cognitive decline by 0.007 and 0.002 standardized units, respectively.
Dhana et al. (2010)	Cognitively normal participants from the Chicago Health and Aging Project (mean age 73.2 ± 5.8 years) and the Rush Memory and Aging Project (mean age 81.1 ± 7.2 years)	6 years	A healthy lifestyle score was defined based on Dietary intake (a 144-item FFQ), light to moderate alcohol consumption, ≥ 150 min/week of physical activity, nonsmoking, and late-life cognitive activities. Employed 0–15 point MIND diet score	Participants with a high lifestyle score (4–5 healthy lifestyle factors) had a 60% lower risk of AD while participants with a moderate lifestyle score (2–3 healthy lifestyle factors) had a 37% lower risk of AD
de la Rubia Ortí et al. (2018)	AD patients institutionalized in the Alzheimer’s Family Association of Valencia (65–85 years old)	21 days	Participants followed an isocaloric MeDi in addition to 40 mL of coconut oil, orally administered for 21 consecutive days. 20–30 g/day of soluble and insoluble fiber was administered to prevent constipation.	Intervention with coconut oil was associated with improvements in episodic, temporal orientation, and semantic memory.

MeDi, Mediterranean diet; FFQ, food frequency questionnaire; AD, Alzheimer’s disease; DELCODE, DZNE-Longitudinal Cognitive Impairment and Dementia Study; CVD, cardiovascular risk; PREDIMED-NAVARRA, Prevención con Dieta Mediterránea (Prevention with Mediterranean Diet); FDG-PET, flurodeoxyglucose-positron emission tomography; PiB-PET, Pittsburgh compound B-positron emission tomography; CMRglc, cerebral metabolic rate of glucose; EVOO, extra-virgin olive oil; MIND, Mediterranean-DASH Intervention for Neurodegenerative Delay; MCI, mild-cognitive impairment; PCA, principal component analysis; BMI, body mass index; DASH, Dietary Approaches to Stop Hypertension.

#### Perspectives on Mediterranean dietary patterns

**FIGURE 3:**
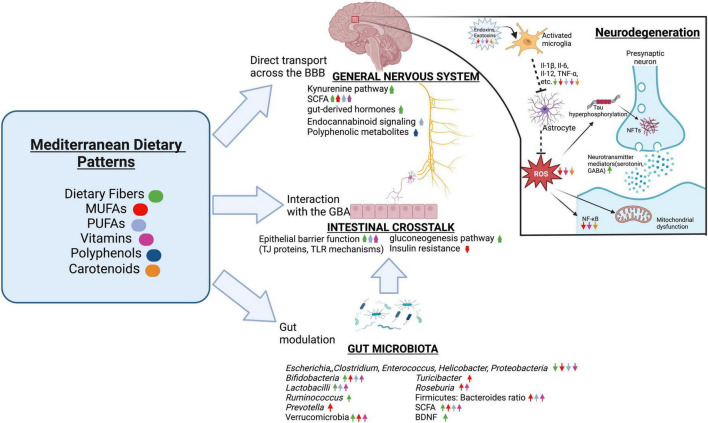
The effects of Mediterranean dietary components on the GBA. Dietary fibers, MUFAs, PUFAs, vitamins, polyphenols, carotenoids, and other bioactive compounds have been found to stimulate SCFA-producing organisms while inhibiting pathogenic bacteria, reducing endotoxemia and inflammatory pathways such as NF-κB, protecting gut barrier integrity independently of the gut microbiota, and promoting the production and translocation of neuroactive molecules across the tightly regulated BBB to directly influence the CNS. Additionally, many components of the Mediterranean dietary pattern neutralize oxidative stress and protect against apoptosis. The color of the arrows correspond to one of the six dietary components, where an up arrow represents the promotion of a factor of the GBA while a down arrow represents the inhibition of said factor. SCFA, short-chain fatty acid; BBB, blood-brain barrier; BDNF, brain-derived neurotrophic factor; TJ, tight junction; TLR, toll-like receptor; MUFAs, monounsaturated fatty acids; PUFAs, polyunsaturated fatty acids; LPS, lipopolysaccharide; IL-1β, Interleukin 1-beta; TNF-α, tumor necrosis factor-alpha; NO, nitric oxide; ROS, reactive oxygen species; GABA, gamma aminobutyric acid; NF-κB, Nuclear factor-κB; GBA, gut-brain axis. We have obtained the appropriate licenses from BioRender for this figure.

While clinical applications of potential dietary pathways are yet to be fully justified, several studies indicate that healthier diets focusing on neuroprotection through specific nutrients and vitamins (e.g., docosahexaenoic acid and other omega-3 fatty acids, vitamins B complex and E, and a lower sodium consumption), physical exercise, and a healthy lifestyle profile (Smith, 2019) could initiate a cascade of metabolic alterations in the gut microbiota, which suppress inflammation and oxidative stress (Sano et al., 1997; Dhana et al., 2010; Yurko-Mauro et al., 2010; Kim et al., 2013; Morris et al., 2017; Moore et al., 2018; Rocaspana-García et al., 2018; Faraco et al., 2019) as depicted in Figure 3. As these lifestyle factors are easily modifiable by the individual, it is imperative to promote feasible strategies consisting of healthy, nutrient-rich diets and lifestyle behaviors among older adults to delay or prevent AD. Other studies with larger samples and longer longitudinal follow-ups are needed to replicate these research findings in community-based samples with more diverse economic, medical, and social backgrounds, as well as to determine whether AD biomarker changes are predictive of AD onset in relation to healthy diet adherence and exposure time (Berti et al., 2018).

### High-fat diets

In contrast to probiotics, fermented foods, MIND/MeDi diets, and other plant-based dietary patterns, the Western diet (WD) is known to promote diverse forms of inflammation and disease through gut modulation (Nagpal et al., 2019; Rusek et al., 2019). The WD is characterized by a high protein content from fatty domesticated and processed meats, saturated fats, refined grains, sugar, alcohol, salt, and corn-derived fructose syrup obtained from common foods (Nagpal et al., 2019; Versele et al., 2020). Major hallmarks of the WD include the ultra-processing of foods and the breakage of cell walls in fiber-rich foods, the use of food additives and noncaloric artificial sweeteners by the masses (emulsifiers have pro-inflammatory potential, while lecithin has the opposite effect, prompting inquiry regarding the role of food additives), and increased amounts of readily accessible acellular nutrients in the small intestine and the colon, which facilitates the growth of certain bacteria specialized in metabolizing simple sugars and promoting pathogen-associated molecular patterns (Zinöcker and Lindseth, 2018). Due to increased territorial competition, the tendency to favor foods providing microbial and human cells with more easily digestible substrates usually implies the reduced intake of cellular plant-based foods and, consequently, the diminished growth of bacteria that degrade fiber and produce beneficial metabolites (e.g., SCFAs) (Statovci et al., 2017). These observations indicate that diet-induced dysbiosis, metabolic disorders, and neuroinflammation are interconnected.

#### Alzheimer’s disease biomarkers

In cross-sectional and longitudinal studies, higher intake of fruit, vegetables, whole grains, oily fish, low-fat dairy, and relevant vitamin supplements (e.g., B12, folate, D) was associated with a reduction in AD biomarkers, such as Aβ40 and Aβ42, tau levels in the CSF, and chemical compounds used in PET imaging, while a diet consisting of high glycemic, high saturated fat foods was associated with an increase in the said biomarker burdens (Hill et al., 2019). Additionally, the WD may augment tau phosphorylation. After treating C57BL/6NHsd mice with a high-fat diet, Kothari et al. (2017) observed an increased expression of the active form of GSK-3β, hyperactivation of Akt and AMPKSer485 through insulin resistance (which inhibits tau dephosphorylation), and a significant decrease in Activity Regulated Cytoskeleton-associated protein, which is crucial in long-term memory consolidation. In turn, the loss of synaptic plasticity through the formation of Aβ plaques and NFTs promotes neuroanatomical changes. Interestingly, adherence to the WD may increase gray matter volume and cortical thicknesses in temporoparietal regions, supporting mounting evidence that early, preclinical increases in cortical volumes foreshadow the pathogenesis of AD later in life because such developments may indicate neuroinflammation (Frye et al., 2021). The mechanisms underlying increased cortical volumes in the context of AD progression require further study to better differentiate between resilient phenotypes and signs of vulnerability, allowing intervention to occur before mild-cognitive impairment (Christensen et al., 2013; Mancuso et al., 2014; Frye et al., 2021).

#### Endotoxins

The WD exhibits an adverse effect on the CNS by upsetting barrier integrity and the balance of gut metabolites, causing chronic activation of glial cells and increasing AD biomarkers (Naja et al., 2015; Statovci et al., 2017). Studies comparing the effects of high-fat WD against plant-based MeDi on AD models (i.e., C56BL/6J mice, APPswe mice, and APP21 transgenic rats) treated with LPS and/or subjected to contextual fear conditioning discovered overall significant differences in brain neuroinflammation status and cognitive testing results between diet groups, with mice consuming the WD exhibiting more cognitive decline during open-field testing (Ivanova et al., 2020; Munster, 2020; Wiȩckowska-Gacek et al., 2021). These findings reveal that mice fed MeDi were better protected against cognitive deficits and AD-related pathologies induced by LPS treatment in the gut and the presence of human APP, including the strong enhancement of full-length APP levels and cleavage of its C-terminal forms, the increased presence of soluble Aβ42, WD-induced hippocampal astrogliosis, and widespread white matter microgliosis, than mice fed WD (Kahn et al., 2012; Ivanova et al., 2020; Wiȩckowska-Gacek et al., 2021).

#### Genetic alterations

Persistent adherence to a high-fat diet aggravated AD-like phenotypes and TREM2 levels in APP23 mice, principally through the altered expression of genes involved in immune responses, nervous system development, synaptic transmission, mitochondrial biogenesis, and apoptosis regulation (Nam et al., 2017). The complementary effects of these gut-modulatory processes relating to mitochondrial dysfunction, enhanced inflammatory responses, increased deposition of Aβ, and the reduced expression of genes related to neuronal projection contribute to the neurotoxic presence of Aβ plaques, which induces behavioral changes reflected in AD models (Nam et al., 2017; Ivanova et al., 2020).

#### Perspectives on western dietary patterns

Ultimately, diets can be considered a nonpharmaceutical tool to accentuate cognitive performance in racially diverse populations (Nutaitis et al., 2019). However, diets lacking fermentable fibers in favor of foods high in saturated fats and virulence potential have become alarmingly commonplace (Rakhra et al., 2020). In addition to metabolic syndrome, which is found to be contemporaneous with the progression of neurodegeneration in AD models, WD strongly accelerates the AD pathological processes through LPS translocation, BBB impairment, intensification of the amyloidogenic APP cleavage path, and chronic activation of glial cells with impaired phagocytic abilities (Ivanova et al., 2020). Thus, the WD should be considered a vital modifiable risk factor of AD development. Accordingly, society must aim to provide better dietary advice on how to better nourish both the gut microbiota and the host.

## Discussion

It is unclear whether most established physiological dysfunctions are causative factors of AD, results of AD, or both. According to the existing body of literature, AD pathogenesis is triggered by the interdependent reverberations of Aβ plaque formation, hyperphosphorylation of tau protein, NFT accumulation, glial inflammation, and the ultimate loss of neural plasticity and proper neuron function. The search for nonpharmaceutical tools to relieve AD symptoms sparked interest in gut-modulatory therapies, which are expected to have a considerable influence on brain homeostasis through the GBA.

The GBA allows microbes, metabolites, and neuroactive compounds to communicate *via* a complex network of neural, hormonal, and immunological pathways. As a result, dietary interventions help reverse increased oxidation levels, impaired neuroactive pathways, and damage to the HPA axis, conferring health benefits that could prove helpful in managing AD. Fixing diet-induced dysbiosis is an especially intriguing first step toward addressing the AD epidemic, as current FDA-approved medications have proven to be ineffective and emphasize the need for alternative approaches. Indeed, several researchers speak of the promising potential of gut microbiota-modifying therapies, such as probiotics, fermented foods, and plant-based dietary patterns. Inevitably, eliminating Western foods and unhealthy habits may also reverse the progression of neurodegenerative diseases.

Yet, while research attention has focused on the potential ameliorating role of diet in neurodegeneration, there has been little evidence underscoring both disease-modifying/total reversal capabilities as well as the practicality of an all-encompassing diet therapy for the clinical management of AD, especially in late-onset stages. Thus, significant lifestyle changes should be considered as purely a preventative measure for AD in the healthy population, and this quality could be made a priority during stages of mild cognitive impairment to help alleviate stresses on the GBA. This is especially vital considering the feasibility of simple dietary switches before and during the early stages of AD. Research on cases of severe cognitive decline should review the plausibility of dietary adherence in the context of medication side effects, a decline in appetite, the need to shift focus to the prevention of malnutrition (which entails a high-calorie diet rather than specific dietary adherence), and the consideration that aggressive nutritional support may not be appropriate once significant neurodegeneration has occurred. These concerns raise the need to identify the exact period in late-stage AD when it becomes medically futile to take dietary precautionary measures.

Future studies should continue to compare the longitudinal effectiveness of different dietary patterns as a strategy to reverse AD during progressive stages, identify the relative sequence and timing of such dietary interventions within a very complex cascade of physiological events with the assistance of preclinical biomarkers, and help establish future dietary guidelines that promote neuroprotective foods and augment brain function.

## Author contributions

DL, VL, and SH: conceptualization. DL: writing—original draft preparation. DL and SH: writing—review and editing. VL and SH: funding acquisition. All authors have read and agreed to the published version of the manuscript.
